# Supplementary figures and images for: Dye-enhanced visualization of rat whiskers for behavioral studies
Source: eLife. 2017 Jun 14;6:e25290. doi: 10.7554/eLife.25290 (PMC5511012; doi:10.7554/eLife.25290)

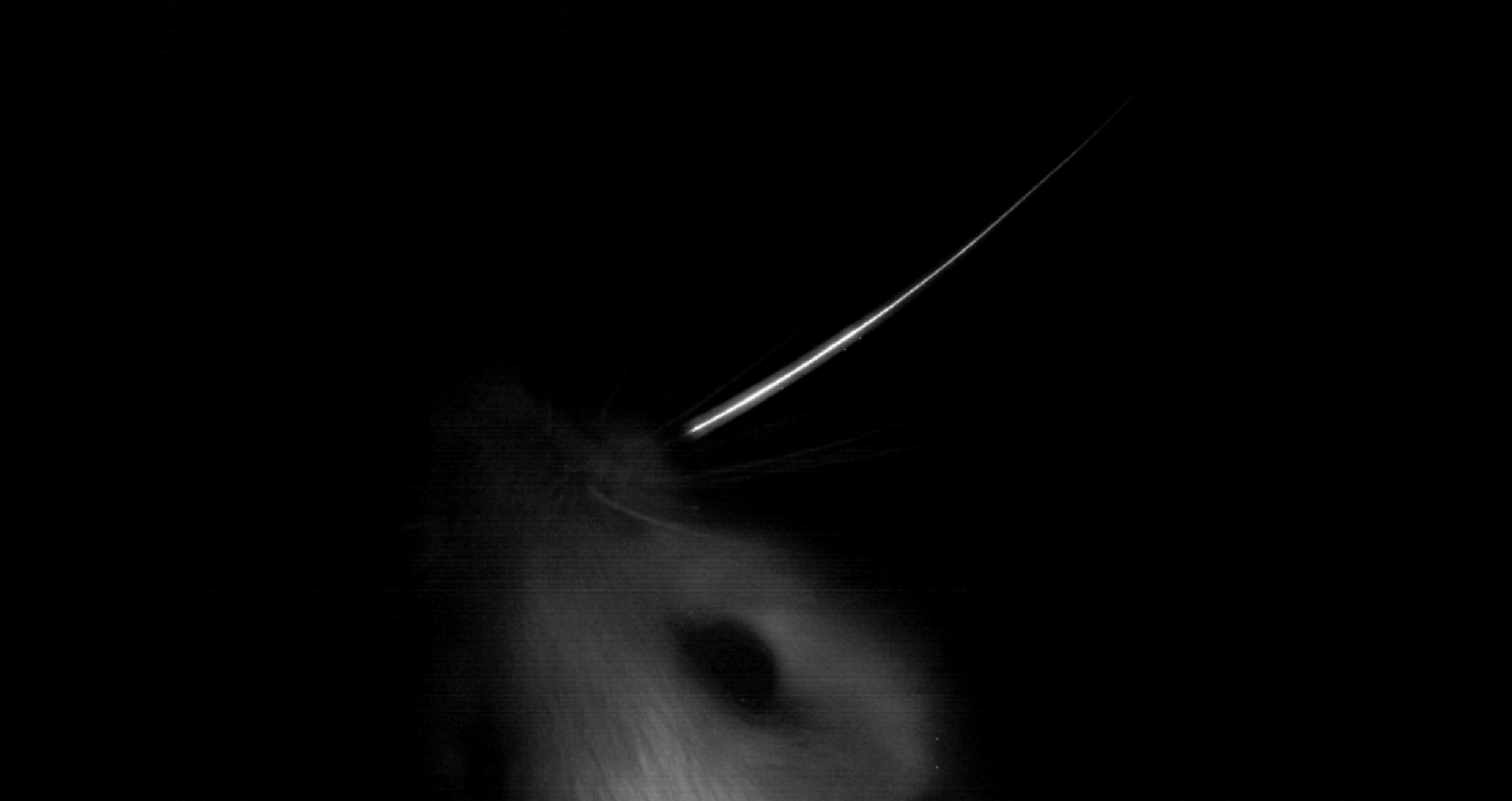

Supplement: Supplementary file 1. — A water reward spout armed with a licking sensor triggered the high-speed video recording (this is a frame of Video 2). The field of view is illuminated with four out of seven of the lamp’s LEDs and an orange plexiglass is used as an optical filter, demonstrating a high enough signal-to-noise ratio to allow whisker tracking, which is overlaid. DOI: http://dx.doi.org/10.7554/eLife.25290.008 [file elife-25290-supp1.pdf]

**A**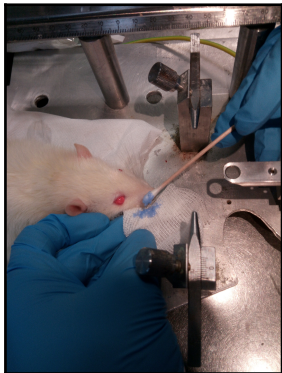**B**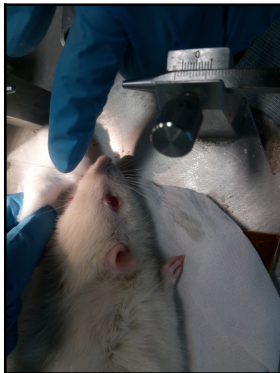**C**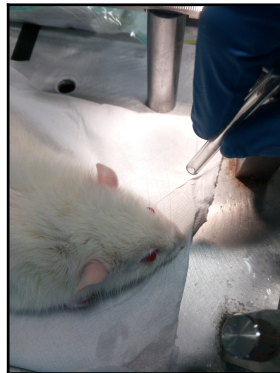**D**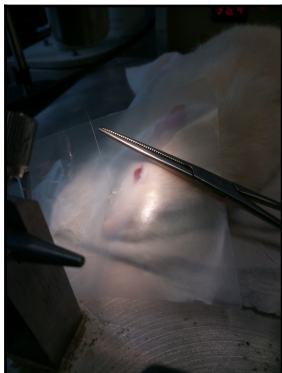**E**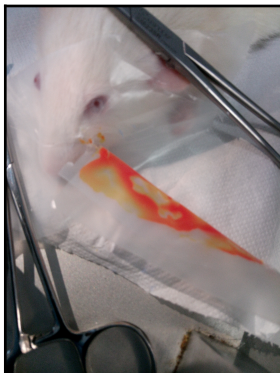**F**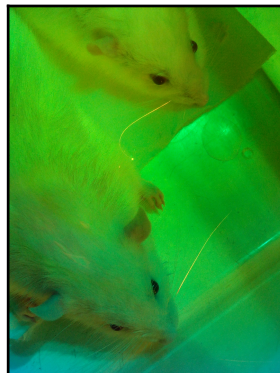

Supplement: Supplementary file 2. — After sedation with Domitor: (A) all whiskers are chemically bleached. (B) The bleaching factor is removed with wet cotton and (C) dried with airflow. (D) The single whisker is isolated by paraffin film; (E) color is applied using another paraffin film and after the application time the excess color is washed away as in (B); (F) whisker coloring is tested under the long-pass filter. DOI: http://dx.doi.org/10.7554/eLife.25290.009 [file elife-25290-supp2.pdf]

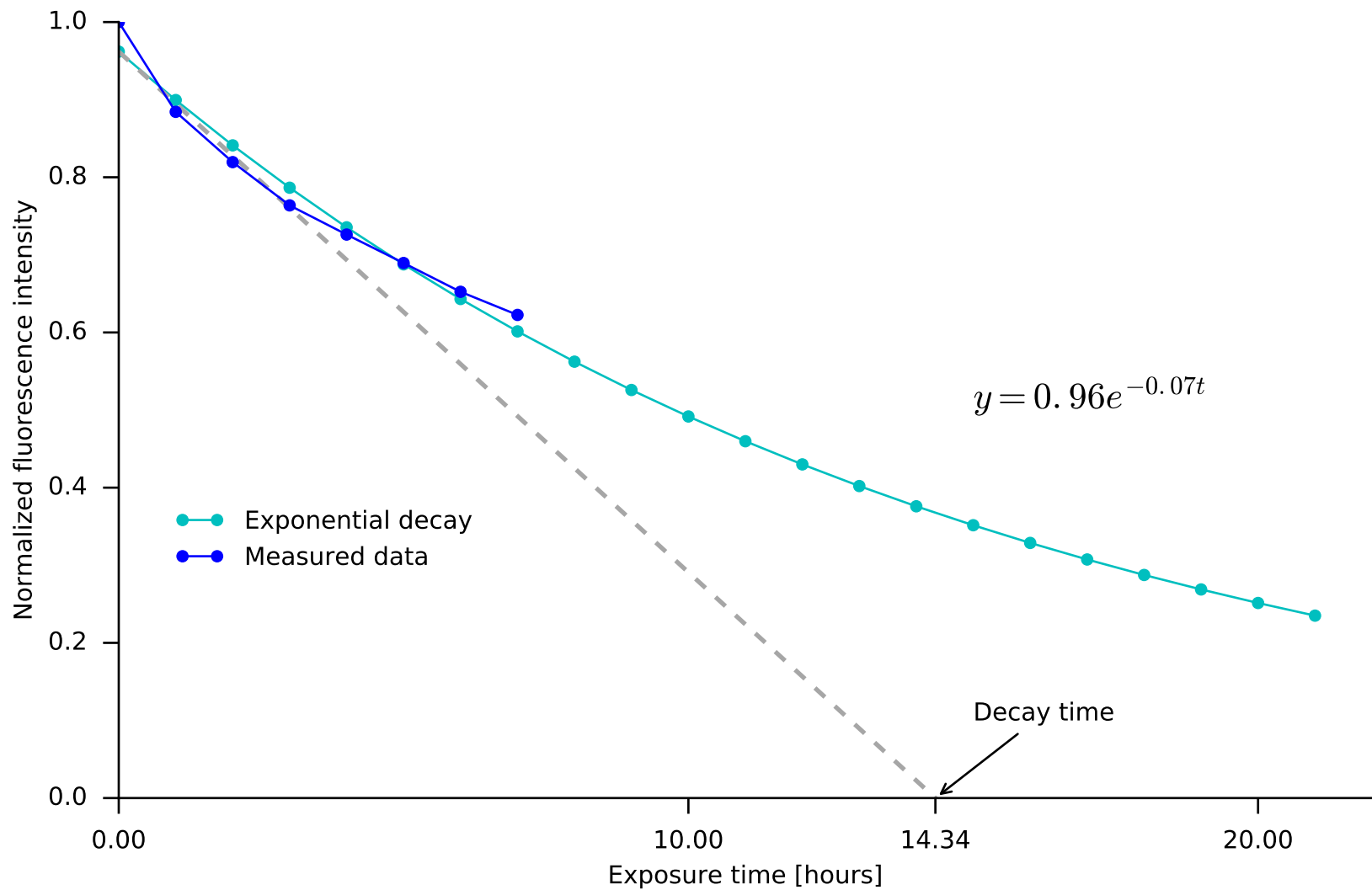

Supplement: Supplementary file 3. — Normalized fluorescence intensity of the whisker (dark blue); exponential decay fit (light blue); exponential time constant expressed in hours of exposition (dashed line intersection with abscissa). DOI: http://dx.doi.org/10.7554/eLife.25290.010 [file elife-25290-supp3.pdf]
