# Supplementary material for: Dye-enhanced visualization of rat whiskers for behavioral studies
Source: eLife. 2017 Jun 14;6:e25290. doi: 10.7554/eLife.25290 (PMC5511012; doi:10.7554/eLife.25290)
Supplement: Supplementary file 4. — Recall that the modal frequencies ωi do not depend on the Young’s modulus and mass density independently, as they depend on their ratio. This can be inferred from Equation (1) or Equation (5) in Materials and methods. Hence, we have tuned this ratio to fit the experimental results by keeping fixed the mass density and changing the Young’s modulus. DOI: http://dx.doi.org/10.7554/eLife.25290.011 [file elife-25290-supp4.docx]

**SUPPLEMENTARY MATERIALS**

| $L\left[ mm \right]$ | $r_{b}\left[ \mu m \right]$ | $r_{t}\left[ \mu m \right]$ | $E/\rho\left[ GPa{m^{3}}/{kg} \right]$ | $\alpha\left[ 1/s \right]$ | $\beta\left[ s \right]$ |
| --- | --- | --- | --- | --- | --- |
| 56 | 94 | 7 | 3.77x10^3^ | 50 | 1.5x10^-4^ |
| 53 | 95 | 6 | 2.89x10^3^ | 70 | 1.5x10^-4^ |
| 45 | 81 | 4 | 3.7x10^3^ | 120 | 1.0x10^-4^ |
| 41 | 83 | 7 | 4.39x10^3^ | 30 | 2.0x10^-4^ |
| 38 | 89 | 7 | 2.89x10^3^ | 80 | 1.3x10^-4^ |
| 37 | 67 | 9 | 3.95x10^3^ | 60 | 1.3x10^-4^ |
| 33 | 82 | 6 | 1.58x10^3^ | 80 | 1.2x10^-4^ |
| 26 | 48 | 3 | 4.39x10^3^ | 80 | 4.0x10^-4^ |
| 23 | 49 | 4 | 3.25x10^3^ | 20 | 0.8x10^-4^ |
| 18 | 40 | 3 | 2.63x10^3^ | 50 | 1.0x10^-4^ |

**Supplementary file 4**
